# Raised temperatures over the Kericho tea estates: revisiting the climate in the East African highlands malaria debate

**DOI:** 10.1186/1475-2875-10-12

**Published:** 2011-01-17

**Authors:** Judith A Omumbo, Bradfield Lyon, Samuel M Waweru, Stephen J Connor, Madeleine C Thomson

**Affiliations:** 1 International Research Institute for Climate and Society (IRI), The Earth Institute at Columbia University, LDEO Campus, Palisades, New York, 10964-8000, USA; 2 Kenya Meteorological Department, P. O. Box 30259, 00100, Nairobi, Kenya

## Abstract

**Background:**

Whether or not observed increases in malaria incidence in the Kenyan Highlands during the last thirty years are associated with co-varying changes in local temperature, possibly connected to global changes in climate, has been debated for over a decade. Studies, using differing data sets and methodologies, produced conflicting results regarding the occurrence of temperature trends and their likelihood of being responsible, at least in part, for the increases in malaria incidence in the highlands of western Kenya. A time series of quality controlled daily temperature and rainfall data from Kericho, in the Kenyan Highlands, may help resolve the controversy. If significant temperature trends over the last three decades have occurred then climate should be included (along with other factors such as land use change and drug resistance) as a potential driver of the observed increases in malaria in the region.

**Methods:**

Over 30 years (1 January 1979 to 31 December 2009) of quality controlled daily observations ( > 97% complete) of maximum, minimum and mean temperature were used in the analysis of trends at Kericho meteorological station, sited in a tea growing area of Kenya's western highlands. Inhomogeneities in all the time series were identified and corrected. Linear trends were identified via a least-squares regression analysis with statistical significance assessed using a two-tailed t-test. These 'gold standard' meteorological observations were compared with spatially interpolated temperature datasets that have been developed for regional or global applications. The relationship of local climate processes with larger climate variations, including tropical sea surface temperatures (SST), and El Niño-Southern Oscillation (ENSO) was also assessed.

**Results:**

An upward trend of ≈0.2°C/decade was observed in all three temperature variables (P < 0.01). Mean temperature variations in Kericho were associated with large-scale climate variations including tropical SST (r = 0.50; p < 0.01). Local rainfall was found to have inverse effects on minimum and maximum temperature. Three versions of a spatially interpolated temperature data set showed markedly different trends when compared with each other and with the Kericho station observations.

**Conclusion:**

This study presents evidence of a warming trend in observed maximum, minimum and mean temperatures at Kericho during the period 1979 to 2009 using gold standard meteorological observations. Although local factors may be contributing to these trends, the findings are consistent with variability and trends that have occurred in correlated global climate processes. Climate should therefore not be dismissed as a potential driver of observed increases in malaria seen in the region during recent decades, however its relative importance compared to other factors needs further elaboration. Climate services, pertinent to the achievement of development targets such as the Millennium Development Goals and the analysis of infectious disease in the context of climate variability and change are being developed and should increase the availability of relevant quality controlled climate data for improving development decisions. The malaria community should seize this opportunity to make their needs heard.

## Background

### Climate information needs for malaria

Malaria is a climate sensitive disease and climate information can be used to monitor and predict aspects of its spatial distribution [1,2] seasonality [3] year-to-year variability [4] and longer term trends [5]. Furthermore, climate information is increasingly recognized as necessary to enable accurate impact evaluations of malaria interventions [6,7]. The biology of malaria transmission is markedly complex, involving interactions between multiple, constantly changing, extrinsic and intrinsic factors, many of which cannot be easily measured and are therefore challenging to model. Mathematical models of malaria transmission are highly sensitive to the non-linear response of both the vector and parasite to variations in temperature [8]. Thus, the issue of temperature variability and change is often considered central to the discussion of whether malaria transmission is likely to increase if global temperatures rise [9-11].

### Conflicting evidence from the Kenyan highlands

Here the focus is on the use of climate information to understand the possible impact of climatic trends on increases in malaria incidence in the East African Highlands over the last three decades (1980-2009). For over a decade, the highlands of malaria endemic countries have been considered areas of special concern for the impacts of climate change [12]. Lindsay and Martens suggested in 1998 that, with all other factors remaining equal, global warming may result in the geographic spread of malaria transmission into previously malaria-free highland areas [13]. Since then, the discussion around the evidence for this has raised a heated and highly polarized debate. Multiple peer reviewed publications [14-17], newspaper articles, editorials and blogs have been written and yet, to date, the debate continues to smolder.

**Figure 1:**
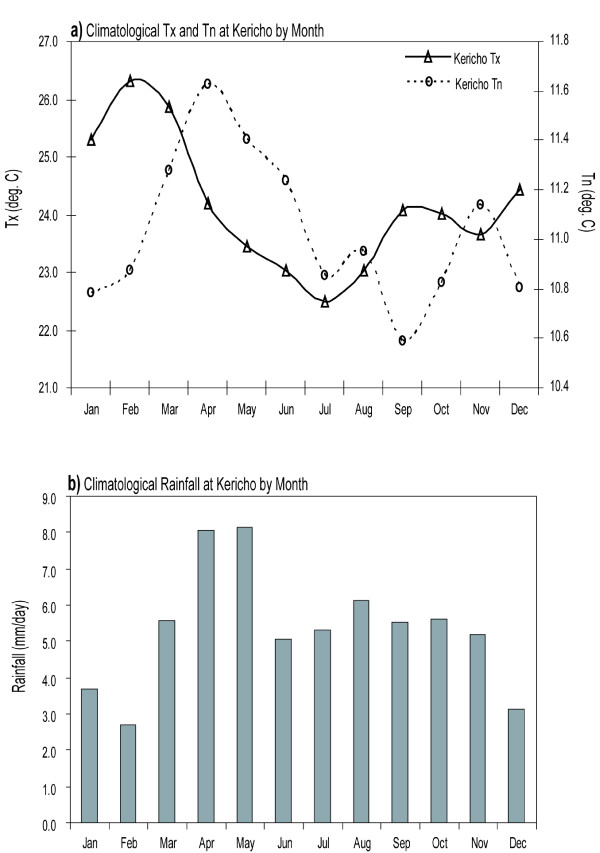
**Average monthly climate conditions at Kericho for the period 1980-2009 a) lines indicate monthly average temperature (deg. C) Tx (left axis) and Tn (right axis) b) bars indicate average monthly rainfall (mm/day, left axis)**.

The controversy has centered on analyses conducted with data relating to the East African highlands and in particular, the tea estates in Kericho, a district that lies at 1600 to 3000 metres above sea level in the highlands west of the Great Rift Valley in Kenya. The region is ideally suited for growing tea which typically does well in a warm climate with well-distributed rainfall (Figure 1). More than 30 years of laboratory confirmed malaria incidence data are available from the Brooke Bond tea estate health facilities from a period where confounding effects due to changes in intervention coverage, demographics and environment are constrained [18]. There is a paucity of long time series of high quality malaria incidence data for highland areas in Africa so the Kericho tea estate data are a significant resource for modeling malaria epidemics and the potential impact of climate change. However, less attention has been given to the quality and relevance of the climate data used in the analyses of temperature and malaria.

To-date research has focused on addressing four key questions regarding the relationship between malaria and temperature in the East African highlands: a) is malaria increasing or re-emerging? b) are temperatures increasing? c) if there is a warming trend in the highlands, is it related to global climate change? and d) if there is a warming trend, is there a causal relationship with trends in malaria incidence? Here the focus is on questions a, b and c; a quality controlled meteorological dataset for Kericho is made available for use in answering question d (see Additional file 1).

### Is malaria increasing?

Detailed epidemiological data for Kericho and elsewhere in the highlands of Eastern Africa provide strong evidence of emergence and resurgence of malaria in the 1980s and 1990s [12,17,19,20]. A supporting study by Malakooti and colleagues [18] shows that the majority of infections of patients attending the Kericho tea estate health facilities between 1990 and 1997 were acquired locally. However recent reinvigorated malaria control efforts may have arrested or reversed this trend in some areas [21-24].

### Are temperatures increasing?

Central to the debate over the last decade has been whether or not a statistically significant upward trend in temperature in the highlands has occurred and whether such a rise could account, at least in part, for the observed increase in malaria. Initial analyses of the malaria data from the Kericho tea estate with contemporaneous monthly averaged meteorological data presented no evidence for any significant changes in climate during the period of study [18,20].

A substantial constraint to the climate analyses of these and subsequent studies has been the very limited access to a sufficiently long time series of quality controlled daily observations of surface air temperature from meteorological stations in Kericho. Constraints to accessing such gold standard observations have meant that studies have relied heavily on limited time series of station data [25], have used data that are inadequately quality controlled [26] or have ignored local ground observations completely in favour of spatially interpolated datasets[27,28]. These latter data are intended for regional or global-scale analyses and utilize only a fraction of the stations that are maintained by National Meteorological Services and thus employ substantial spatial interpolation in their construction often leading to insufficient information for local point scale analyses. These limitations are clearly stated by the developers of the datasets [29].

### Are local climate processes related to global patterns?

A recent climate analysis for East Africa was conducted by Christy and co-authors examining air temperature trends at 60 stations across Kenya [30]. After spatially interpolating the station-based data, the study reports finding a statistically significant upward trend in minimum temperature (Tn) in the Kenyan Highlands region. The magnitude of the area-average trend in Tn they identified was about ^+^0.15°C per decade, based on an analysis covering the period 1979-2004. However, no statistically significant trend in maximum temperature (Tx) was found. Christy *et al *[30] suggest that Tx is a better measure of the climate trend signal, as Tn is more sensitive to local processes including changes in the land surface. The study, however, did not explicitly examine the relationship between temperature variations in highland areas with global scale climate variations.

### Availability of quality controlled climate data

Accessing reliable, quality-controlled meteorological data has been a fundamental challenge in assessing temperature trends in East Africa's highlands, including their relationship to global processes. In particular, although available within national meteorological services, the cost associated with obtaining daily records, is a significant constraint to their use. Full access, quality control and proper use of these data requires the bridging of a number of technical, capacity and institutional gaps [31].

Although significant progress has been made to improve the quality of public domain, gridded meteorological data, their accuracy relies heavily on the quality and consistency of the observation input data used in their development. Where information from ground stations is sparse, the accuracy is necessarily limited. Given the limited access to station observations, many studies of temperature trends in Kericho have based their analyses on different versions of a gridded data set developed by the Climate Research Unit (CRU) of the University of East Anglia (UEA) [14,19,32]. The UEA CRU data are monthly mean values of surface climate variables for global land areas developed primarily for regional or global applications [29]. The trend analysis results from these various studies (and versions of the dataset) to date have been inconclusive.

It is proposed that the lack of agreement regarding temperature trends over Kericho has been largely the result of the inappropriate use of spatially interpolated data over Africa obtained from UEA CRU. Here the quality of these data are examined and compared with analyses of trends using gold standard meteorological observations at Kericho. Three recent versions of the UEA CRU data are used, the most recent one incorporating an additional 4 years of observations; all are examined for temporal consistency. The local, observed climate variations at Kericho are then compared with larger climate variations, including tropical SST and El Niño-Southern Oscillation (ENSO).

## Methods

### Construction of monthly time series and homogeneity tests

Digitized time series of daily maximum (Tx) and minimum (Tn) temperature and accumulated Rainfall (R), constructed by the Kenya Meteorological Department (KMD) for their observing station at Kericho (33.35E, 0.36S), are the primary meteorological data used in the analysis. The available data cover the period 1 January 1979 to 31 December 2009 for Tx and Tn and 1 January 1980 to 31 December 2009 for R. Quality control of these data, performed at the Institute of Meteorological Training and Research (IMTR), Nairobi, included: updating of missing records, verification for new records, and range and consistency checking.

The first step in the analysis was to compute monthly mean values from the daily data for each of the three variables. Monthly means were not computed for months having more than one missing daily observation, resulting in monthly time series for Tx, Tn and P that were 97.1%, 99.2%, and 99.2% complete, respectively, for the analysis period. Following this step, the monthly time series of all three variables underwent a homogeneity test to identify any break-points (jumps) in the time series which can arise from non-climate factors such as changes in station location, instrumentation and instrument exposure. Spurious break-points can substantially influence the analysis of temporal trends in time series and their removal requires making adjustments to the original time series once they have been identified [33,34]. Ideally, the dates of any changes in, for example, station location and instrumentation, would have been recorded in order to facilitate comparisons with the dates of any identified break-points. Here, when statistically significant break-points were identified without such documentation, it was assumed they did not represent true fluctuations in climate and were removed through adjustment of the original time series.

The open source software tool RHtestV3 (written in the R programming language and available at http://cccma.seos.uvic.ca/ETCCDMI/software.shtml) was used to check for break-points in the time series analyzed. This software tool was developed at the Climate Research Division at Environment Canada and is among the set of homogeneity testing methods recognized by the World Meteorological Organization for quality control of climatic time series [35]. Essentially, the software tests the null hypothesis that there are no changes in the mean of a time series (which may, or may not, contain a secular trend) against the alternative hypothesis that such a shift in the mean does occur at a time *k*. There may be more than one break-point in the time series.

Mathematically, for a time series of variable *X*(*t*) the software uses a recursive regression algorithm to test the null hypothesis

X(t)=μ+αt+ε

against the alternative hypothesis

$$\begin{array}{c} X(t)=\mu_{1}+\beta t+\varepsilon \text{(for}t\leq k)\\ X(t)=\mu_{2}+\beta t+\varepsilon \text{(for}k-1\leq t\leq N) \end{array}$$

where t is time, N the total number of observations, μ the mean, α and β constants, and the shift at time k equal to μ2 - μ1 [36]. The RHtestV3 software allows prescribing the confidence level for identifying break-points; here that was set to p < 0.01. Once the adjusted Tx and Tn time series were generated they were used to compute a monthly mean temperature (Tmean) as a third temperature variable where Tmean = (Tx+Tn)/2. The mean temperature was computed as the average of the daily minimum and maximum temperature specifically to enable comparison with previous studies. However readers should be aware that the calculation of the 'true' mean temperature is more complex; ideally requiring 24 hourly measurements [37]. The quality controlled Tx Tn and Tmean monthly data for Kericho is made available (see Additional file 1) with agreement of the Kenya Meteorological Department.

### Trend Analysis of Tx, Tn and Tmean

The adjusted, monthly time series of Tx, Tn and Tmean were examined to see if there have been any statistically significant temporal trends over the period 1977-2009. Linear trends were identified via a least-squares regression analysis with statistical significance assessed using a two-tailed t-test. Since trends need not be linear, the nonparametric Mann-Kendall (MK) test was also used to identify any statistically significant trends without any *a priori *assumption of their form [38]. The MK test also reduces the effect of outliers on the trend analysis.

### Kericho temperatures and their relation the global tropics

To facilitate comparison with other climate variables, monthly departures from the long-term (1980-2009) monthly mean values of Tx, Tn, Tmean and P were computed. To emphasize relationships with El Niño (and La Niña), an 11-month moving average was then applied to one set of the resulting time series as that is the typical time scale of individual ENSO events. Time series of monthly values (i.e., no moving average applied) of all four variables were also retained for analysis with other climate variables, including monthly time series with the linear trend removed. Tropical SST data for the period 1980-2009 were obtained from the 2.5 deg. latitude/longitude resolution Extended Reconstructed Version3b monthly gridded dataset [39]. Monthly departures from the climatological average tropical SST were calculated and averaged over 25˚S to 25˚N, with the resulting time series then used to compute temporal correlations with different variables. A similar time series was constructed using monthly surface land temperature departures from average for the global tropics obtained from the UEATEM3 dataset which is gridded at 5.0 deg. latitude/longitude resolution [40]. When performing t-tests on correlations between climate variables, the degrees of freedom needed to be determined. This was done using the lagged autocorrelation of respective variables [41] where:

$$\text{T}=1+2\overset{n}{\underset{i=1}{\sum }}\rho_{Ai}\cdot \rho_{Bi}^{'}$$

and $N_{dof}=\frac{N_{obs}}{\text{T}}$

In the above expressions ρ_Ai _and ρ_Bi _are the ith month lag autocorrelation of variables A and B, with *n *set to 18 months (or the number of months when the autocorrelation remained positive if less than 18 months). *N_obs _*is the number of monthly values in the full time series and *N_dof _*is the number of degrees of freedom used to conduct the t-test.

### Highland temperature variations based on gridded temperature analyses

The gridded data in this analysis are of 0.5 degree latitude/longitude resolution, derived by spatial interpolation of ground station observations [29]. The data set includes the variables of maximum, minimum and mean temperature, rainfall, diurnal temperature range and vapor pressure. When direct observations of these variables are not available they are derived from other, observed variables. Time series from Versions CRU05, 2.1 and interim Version 3.0 of the UEA CRU data are plotted and investigated for consistency in variability and in trend in Tx and Tn relative to observations from Kericho.

## Results

### Assessment of Kericho meteorological observation station data

**Figure 2:**
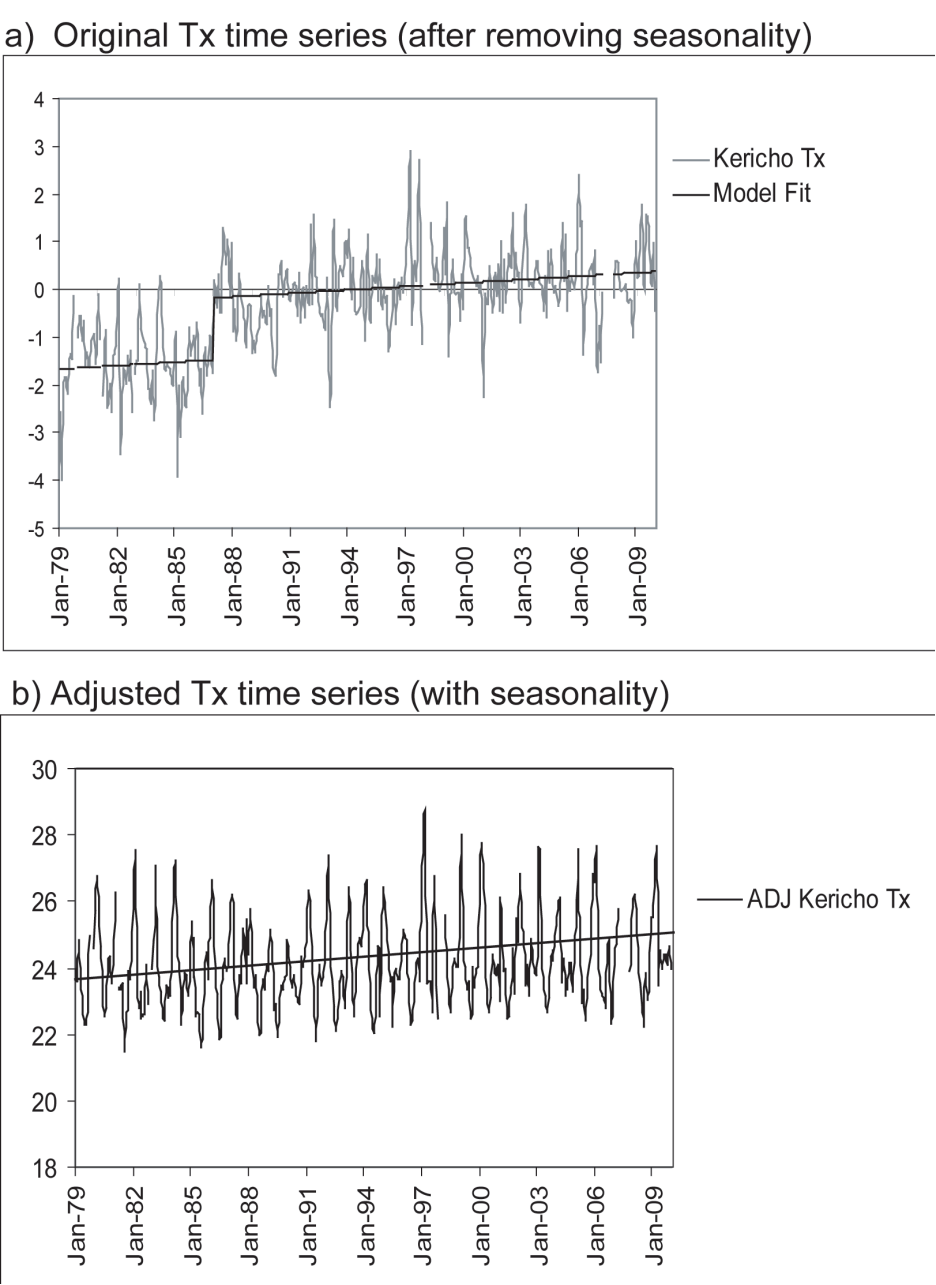
**Time series of monthly values of Tx after removing seasonality (i.e., the 30-year mean values shown in Figure 1) (deg. C) for a) original data, also showing model fit, and b) adjusted data after removing an identified break-point and including seasonality**. The linear trend for the full data period is shown by the solid line (see Table 1 for slope and significance).

**Figure 3:**
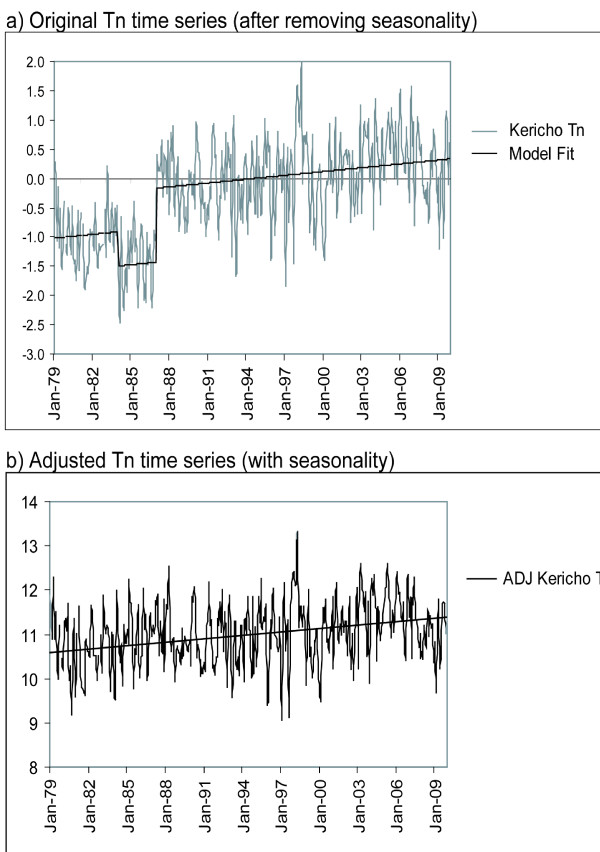
**Time series of monthly values of Tn after removing seasonality (i.e., the 30-year mean values shown in Figure 1) (deg. C) for a) original data, also showing model fit, and b) adjusted data after removing two identified break-points and including seasonality**. The linear trend for the full data period is shown by the solid line (see Table 1 for slope and significance).

In the Tx time series (Figure 2a) a single break-point, with an associated shift of 1.29°C, was detected in 1986. KMD indicates the timing of this shift corresponds to a change in the Kericho station location from the Hail Research Center (35.27E, 0.37S; elevation 2184m) to its current location (35.35E, 0.37S; elevation 1976m) at that time. The sign and magnitude of the identified shift in the mean temperature are both consistent with expectations given the average change in atmospheric temperature with elevation (lapse rate). An adjusted Tx time series (Figure 2b) was generated by matching the means of the two segments of the overall time series, eliminating the break-point. The Tn time series (Figure 3a) also revealed a breakpoint in 1986 (shift of 1.26°C), consistent with the shift in Tx at that time. However, an additional break-point was detected in 1983, with a shift of 0.58°C. There are no documented changes made at the Kericho station at that time but the Tn series was adjusted to remove both of these break-points (Figure 3b). No break-points were detected in the monthly rainfall time series. For the full monthly time series, Tmean, Tx and Tn all exhibited highly significant (p < 0.01) positive trends (0.21°C, 0.24°C and 0.21 °C per decade, respectively, see Table 1).

**Table 1:** Results of trend analysis and significance testing of in Tx, Tn and Tmean*

	Maximum Temperature	Minimum Temperature	Mean Temperature
**Linear trend °C/decade**	0.24	0.21	0.21

**Confidence Level of Trend**	p < 0.01	p < 0.01	p < 0.01

**Trend Range**	0.10 - 0.37	0.11 - 0.31	0.14 - 0.29

*Data obtained from the Kericho meteorological station (33.35E, 0.36S) operated by the Kenyan Meteorological Department.

**Figure 4:**
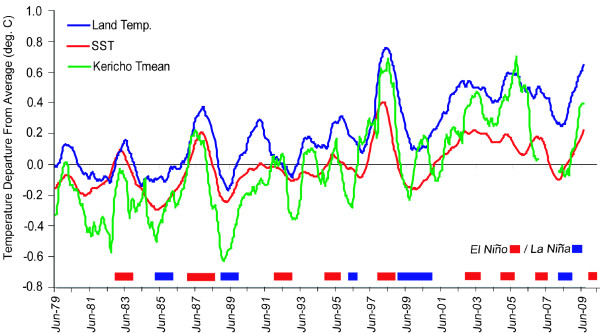
**Time series of monthly departures from 1980-2009 mean values (deg. C) with an 11-month moving average applied for Kericho Tmean (green line), global tropical SST (25S-25N) (red line) and tropical land area mean temperature (blue line)**. Color bars at the bottom of the figure show the occurrence of El Niño and La Niña events (based on the definition in use at the US Climate Prediction Center). See Table 2 for temporal correlations between these variables

**Figure 5:**
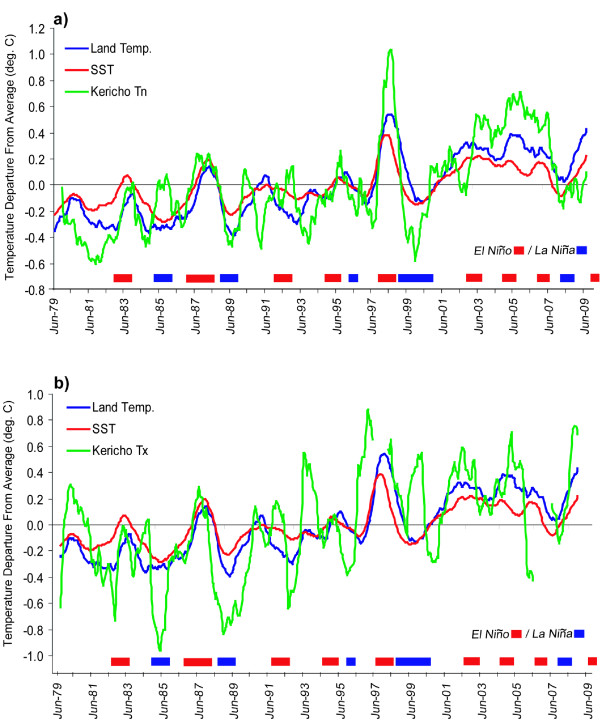
**Time series of monthly departures from 1980-2009 mean values (deg. C) with an 11-month moving average applied for Kericho a) Tn and b) Tx (green line), global tropical SST (25S-25N) (red line) and tropical land area mean temperature (blue line)**. Color bars at the bottom of the figure show the occurrence of El Niño and La Niña events (based on the definition in use at the US Climate Prediction Center). See Table 2 for temporal correlations between these variables

**Figure 6:**
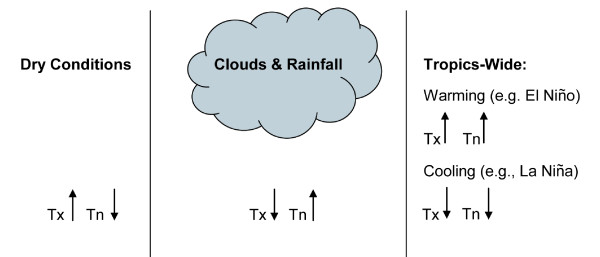
**Schematic of the relationship between Tx and Tn with unusually dry conditions (left), rainy conditions (center) and in relationship to tropics-wide warming or cooling associated with El Niño or La Niña (right)**.

Figure 4 compares the Tmean time series for Kericho with the average tropical (25°S-25°N) SST and land temperature anomalies (departures from the average monthly values for the period 1980-2009). An 11-month moving average was applied to each time series to emphasize variations associated with ENSO. Statistically significant temporal correlations (p < 0.01; see Table 2) are found between Tmean and the two tropics-wide time series. This was the case even when no moving average was applied to the time series. The occurrence of El Niño and La Niña events are typically seen to be associated with warmer and cooler than average values of Tmean at Kericho, respectively. Upward trends in tropical SST and land surface air temperature, consistent with the trend in Tmean at Kericho station, are also seen over this analysis period (c.f. Table 2). Figures 5a and 5b show the same analysis, but for Tn and Tx at Kericho. The influence of El Niño and La Niña is still observed, but Tx is found to behave somewhat differently than Tn. The reason for this difference is the confounding influence of rainfall on temperature that influences Tx and Tn differentially. Months with above-average (unusually heavy) rainfall e.g. during a typical El Niño event, are found to be negatively correlated with Tx values (r = -0.52, p < 0.01) but are positively correlated with Tn (r = 0.30, p < 0.01) (Figure 6). Conversely, unusually dry conditions (e*.g*. during a typical La Niña) boost Tx while allowing Tn to drop. This is an essential point when attempting to link climate variability with health outcomes: care is needed in choosing the appropriate climate variable to analyze. Often average temperatures (mean of Tx and Tn) are used in such analyses, which may be less than optimal given this effect.

**Table 2:** Temporal correlations between climate variables*

Variables	Correlation
Tropical SST, Tropical Land Temperature	0.88 *** 0.77 *** 0.66 ***

Tropical Land Temperature, Kericho Tmean	0.87 *** 0.56 *** 0.41 ***

Tropical SST, Kericho Tmean	0.82 *** 0.50 *** 0.35 ***

Tropical Land Temperature, Kericho Tn	0.78 *** 0.44 *** 0.33 ***

Tropical Land Temperature, Kericho Tx	0.67 *** 0.32 *** 0.22 ***

Tropical SST, Kericho Tn	0.77 *** 0.46 *** 0.36 ***

Tropical SST, Kericho Tx	0.62 *** 0.24 *** 0.14 *

*Confidence levels, based on a 2-tailed t-test, are shown by asterisks, where *** = 99%, ** = 95% and * = 90%. The three correlation values for each pair of climate variables are those computed for: (top) the 11-month moving average; (middle) monthly values; and (bottom) de-trended, monthly values.

### Assessment of the UEA CRU data

**Figure 7:**
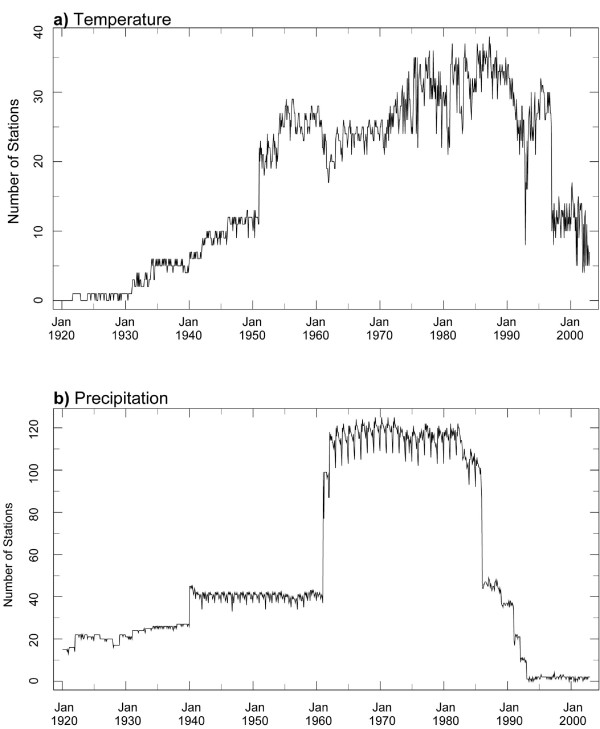
**Number of stations reporting temperature observations (a) and rainfall observations (b) in UEA CRU v2.1 that are within the "radius of influence" of the grid point closest to the Kericho station. Monthly values, 1920-2002**.

For much of Africa, reporting from meteorological stations has been inconsistent in time. More specifically over Kenya, the availability of station data has varied markedly over the last century. Figure 7 shows the number of stations within the "radius of influence" from the grid point closest to the Kericho station in the UEA CRU v2.1 dataset as identified by the developers of the dataset. Temperature recording stations (Figure 7a), for example, were limited to < 10 stations before the 1950s, increasing until 1990s and since then there has been a marked decline of observations. The picture is similar for rainfall observations (Figure 7b) with the maximum number of stations reporting between 1960 -1980 and an almost complete collapse in reporting in more recent years. Although the variable vapour pressure (VP) is available in the dataset, it is noteworthy that there have been no direct observations of VP at all in the entire period (1920-2002). Values of this variable are based entirely on parameterization with other, observed variables, which does not account for local factors which may substantially affect such parameterizations. Clearly, the low density of stations, paucity of observations and irregularity of reporting are considerations for the rationale for using these data to understand local climate processes and particularly longer-term trends.

**Figure 8:**
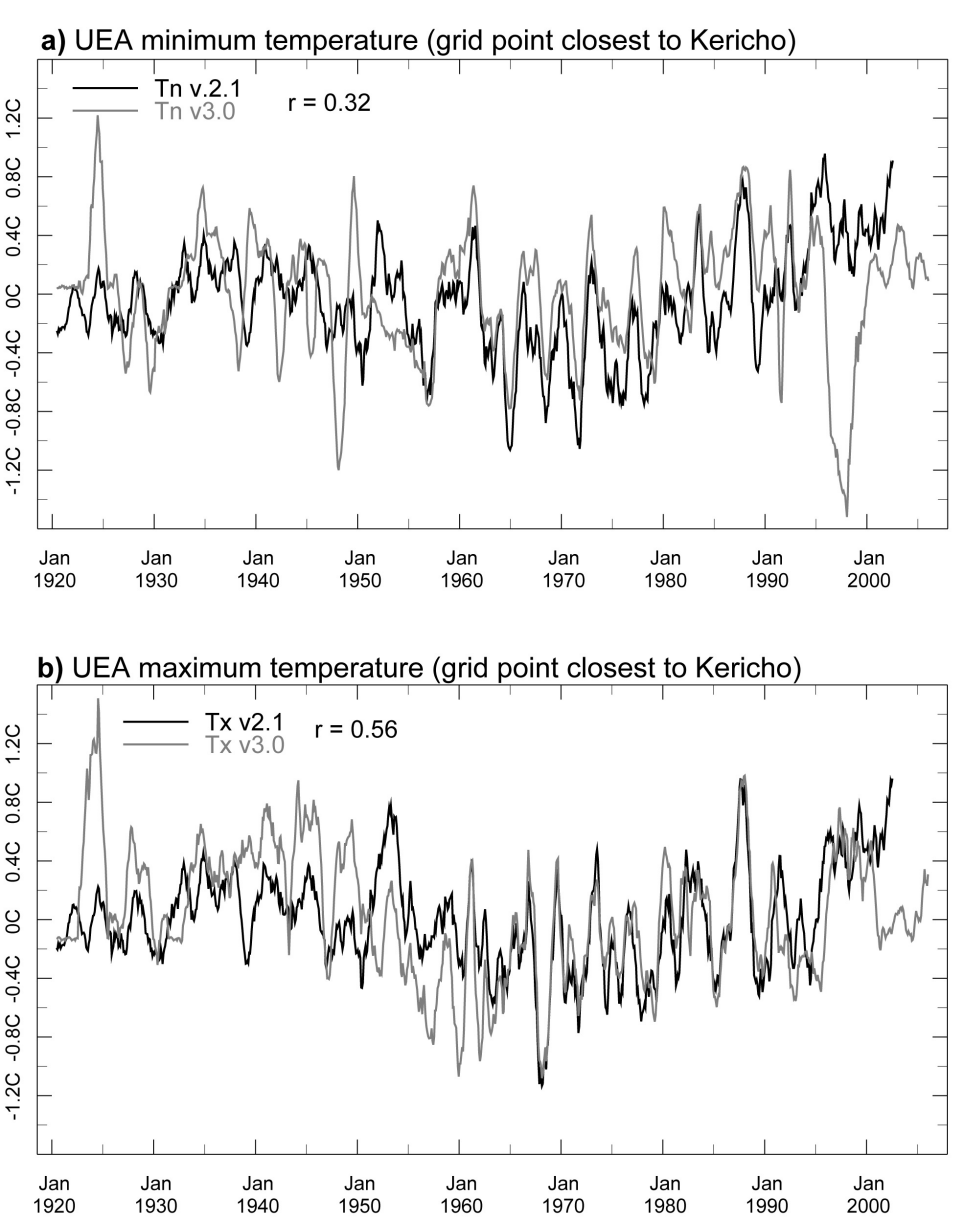
**Time series (1920-2002) of Tn (a) and Tx (b) of the departures from the 1971-2000 average monthly values (an 11-month moving average applied) for versions 2.1 and interim 3.0 of the UEA CRU data and the gridpoint closest to the Kericho station location**. The temporal correlation between the two series is shown in the upper-left of both panels.

**Figure 9:**
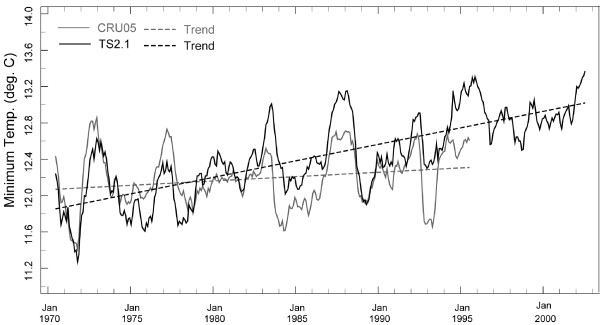
**Time series of Tn based on versions CRU05 and v2.1 of the UEA CRU data for the period 1970 to 1995 (CRU05) and 2002 (v.2.1)**. Best-fit linear regressions are shown as dashed lines for the two versions, which show marked differences.

An additional analysis compares two versions of the UEA CRU data (v2.1 vs. interim v3.0). Figure 8 shows the departure from the monthly average temperature (1971-2000) for the grid point data closest to the Kericho station for these two versions of the dataset (an 11-month moving average applied). The temporal correlation between the two minimum temperature time series is r = 0.32; for Tx it is r = 0.56. The differences in these two dataset versions are likely related, at least in part, to different input stations over the time period analyzed. Such differences can result in markedly different trends. Consider, for example, the linear trends computed for the two Tn time series shown in Figure 9. The two versions of the UEA CRU data shown here (CRU05 and v2.1) are the same temperature data sets used in analyses of temporal trends at Kericho [19,32]. Both time series show the monthly departures from the 1971-2000 average with an 11-month moving average applied. The data for CRU05 ends in 1995 and for v2.1 in 2002. The trend line for v2.1 has a positive slope while interim CRU05 shows essentially no trend.

### Discussion

Initial analyses of the Kericho tea estate malaria data with contemporaneous local monthly averaged meteorological observations recorded at the centre of the estates by the Tea Research Foundation, found no evidence of any significant changes in climate during the period of epidemiological transition [18,42]. The authors concluded that drug resistance, rather than climate change, was the likely key driver of the increasing trend. Furthermore, Fourier analysis of an extended version of the same data sets was used to infer that the observed inter-epidemic period of malaria incidence at Kericho was best explained as function of intrinsic population dynamics rather than cyclical changes in temperature or rainfall[19,43]. Including the Southern Oscillation Index (the atmospheric component of ENSO) in the analysis did not change the result. Subsequent studies by the same group of authors, reinforced by the use of data extracted from the UEA CRU gridded data set (see above) provided additional support for this perspective[14,19,20,27,28] and led these authors to conclude that '*analyses of malaria time-series at sites such as Kericho have shown that malaria incidence has increased in the absence of co-varying changes in climate*.'[14]. It is proposed that the extensive use of the UEA CRU data set to examine temperature trends in Kericho and surrounding areas, despite the data providers indication of the unsuitability of the data for this purpose [29,44] has been central to controversy with regard to evidence for warming or otherwise in the East African highlands. Some authors have misconstrued these results to be based on high quality meteorological records and as a result have concluded that climate cannot be considered an important factor in changes in the frequency or intensity of malaria epidemics in the Kenyan highlands. For example, in a 2008 review article on malaria and global warming in this journal, Reiter [16], referred to the Kericho analysis and, citing Hay *et al *[19], wrote "*Moreover, a set of well-maintained meteorological records shows no significant change in temperature over recent decades*." Pascual and colleagues [32] reported that they had utilized an updated version of the UEA CRU dataset (interim v3.0) to perform an analysis of trends in temperature at the same grid point locations as in the 2002 Hay *et al *study [19] and concluded that there were, in fact, statistically significant positive trends at four locations around Kericho. In a more recent publication, Alonso *et al *[45] drove a malaria model with a mean daily temperature data series created by dovetailing the records from two meteorological stations within the Kericho tea estates, together with adjustments for altitude based on mean temperature data from a number of stations in Kenya spanning a broader altitude range. Predicted malaria cases were found to exhibit a highly nonlinear response to warming, with a significant increase from the 1970s to the 1990s, although typical epidemic sizes are below those observed. The authors proposed that a more detailed analysis using daily minimum and maximum temperature was warranted. Although unable to access a single time series of meteorological data, their result (0.3°C warming over 30 years) is only just outside of the confidence interval (0.14 - 0.29) for mean temperature obtained in the current analysis as presented in Table 1.

A detailed comparison of the UEA CRU data set used in these studies (Figure 9) reinforces the perspective of the data providers regarding the lack of suitability of this data set for local analysis [29]. This study highlights the importance of knowing the origins of the climate data, the need for quality control as well as an understanding of the mechanisms by which local climate variability and change relate to larger climatic processes, before inferences are made. Such an approach has been critical in the evaluation of Kericho temperature trends presented here. Using gold standard meteorological information, the current study has shown clear evidence of an increasing warming trend in Tn, Tx and Tmean over Kericho between 1979-2009. These results are partially supported by the recent study by Christy and colleagues; however the present study differs in that a significant positive trend was also found in Tx. Furthermore the highly significant (p < 0.01) positive trends (0.21°C, 0.24°C and 0.21°C per decade for Tmean, Tx and Tn respectively) presented here were found after careful correction of the time series, a process which will have reduced rather than exaggerated any previously reported trends in the Kericho data set.

While it is possible that local factors may be contributing to the upward trend in Tx at Kericho (as suggested for Tn in the multi-station analysis of Christy *et al *[30]), temperature variations at Kericho in this study were found to be consistent with those in the global tropics (Figures 4 and 5, and Table 2). This indicates that both local and large-scale climate variations are likely at work at Kericho and gives additional validity to the finding of an increasing trend in temperatures at this site.

## Conclusions

Climate is one of many potential drivers of malaria that are measured outside of the health sector. What makes climate measurements unique is the fact that they are recorded according to globally recognized standards at defined, regular time intervals and can be systematically analyzed at the local and global scale allowing comparison across geographical sites and over extended time periods. The fundamental characteristics of climate including its climatology, seasonality, diurnal rhythm and potential predictability at multiple timescales (weather, seasonal, decadal and climate change), make it ideal as an additional layer of information for the health sector for application in malaria vulnerability assessment, surveillance and forecasting. It is argued here that there is potential benefit of the additional information provided by climate data in understanding the epidemiological characteristics of malaria within the changing global environment. A prerequisite is relevant quality controlled climate and epidemiological data.

This study demonstrates that a lack of access to quality controlled meteorological data has substantially undermined the quality of the climate and malaria analyses undertaken to-date. The positive trends in all three climate variables (Tmean, Tx and Tn) suggest that climatic factors in Kericho should not be dismissed as a potentially significant driver of variability and trends in malaria incidence on the erroneous assumption that warming has not taken place. The significance of the warming trend observed in this study ( > 0.2°C per decade) to changes in malaria transmission potential has yet to be assessed but indications from other studies suggest that in this region, even a modest change in temperature can have a significant effect on transmission [11,45]. The missing links in addressing knowledge gaps of the role of local climate processes in disease transmission are high quality data coupled with the skilled interpretation of epidemiologists working in collaboration with climate scientists.

New opportunities exist for improved management of climate-related health risks including malaria. To make use of these opportunities the health community must establish collaborative partnerships with climate/environment research and service communities, work to overcome policy and institutional barriers and identify opportunities for the effective use of climate information in health policy and decision-making. Likewise the meteorological and climate community need to take steps to provide effective, policy relevant climate information and services for the health sector. The Global Framework for Climate Services, the key outcome of the World Climate Conference III, in September 2009 [46] along with several African initiatives including the Climate for Development in Africa (ClimDev-Africa) Programme [47,48] are seeking to address this call for readily accessible meteorological data to support development initiatives. Services should be tailored to appropriate demand and the health community should therefore make their needs heard [49].

## Competing interests

The authors declare that they have no competing interests and that they have all read and approved the final version.

## Authors' contributions

MCT and SJC conceived the analysis. JAO and SMW established the collaboration, obtained the meteorological data, undertook initial analysis and obtained clearance for the distribution of the dataset from KMD. BL undertook the detailed analysis of the meteorological and climate data. JAO, BL and MCT drafted the paper. MCT, SJC, SMW, BL and JAO reviewed the paper and all authors approved the final version.

## Supplementary Material

Additional file 1 **Supplemental Analysis and Monthly Data for Kericho**. Contains analysis of temporal trends in seasonal temperatures and maximum daily values within each month (Table S1) along with examples of the relationships between Tx, Tn and (Figures S1-S5). Click here for file
