# Supplementary material for: Raised temperatures over the Kericho tea estates: revisiting the climate in the East African highlands malaria debate
Source: Malar J. 2011 Jan 17;10:12. doi: 10.1186/1475-2875-10-12 (PMC3031277; doi:10.1186/1475-2875-10-12)
Supplement: Additional file 1 — Supplemental Analysis and Monthly Data for Kericho. Contains analysis of temporal trends in seasonal temperatures and maximum daily values within each month (Table S1) along with examples of the relationships between Tx, Tn and (Figures S1-S5). [file 1475-2875-10-12-S1.DOC]

**Raised temperatures over the Kericho Tea Estates: revisiting the climate in the East African highlands malaria debate**

**Supplemental Information**

Judith A Omumbo^1^, Bradfield Lyon^1^, Samuel M Waweru^2^, Stephen J Connor^1^, Madeleine C Thomson*^1^

^1^International Research Institute for Climate and Society (IRI), The Earth Institute at Columbia University, LDEO Campus, Palisades, New York, 10964-8000, USA.

^2^Kenya Meteorological Department, P. O. Box 30259, 00100, Nairobi, Kenya.

Email: Judith A. Omumbo - [jomumbo@iri.columbia.edu](mailto:jomumbo@iri.columbia.edu); Bradfield Lyon - [blyon@iri.columbia.edu](mailto:blyon@iri.columbia.edu); Samuel M. Waweru - [waweru59@yahoo.com](mailto:waweru59@yahoo.com);

Stephen J. Connor - [sjconnor@iri.columbia.edu](mailto:sjconnor@iri.columbia.edu); and Madeleine C. Thomson - [mthomson@iri.columbia.edu](mailto:mthomson@iri.columbia.edu).

* Corresponding author

**Seasonal trends in minimum, maximum and mean temperature**

Although statistically significant temporal trends were identified for maximum (Tx), minimum (Tm), and mean (Tmean) temperatures in the main text when examining the full time series (i.e., including all months), it is of interest to examine which seasons contribute to those trends. This analysis is described here. First, 12 overlapping, 3-month seasons were defined and identified as JFM for January-March, FMA for February-April, etc. The adjusted, monthly time series of all three temperature variables were then averaged over each of these 12 seasons for the period 1979-2009. The Mann-Kendall test was then applied separately to each of the 12 resulting time series to test for statistical significance. In the implementation used here, the Mann-Kendall test provides a standardized normal variate Z-statistic to examine the probability of that such a value would be obtained by chance. The sign of the Z-statistic represents the sign of the associated trend (which need not be linear) with positive values associated with an increasing trend and negative values with a decreasing trend over the period 1979-2009. A similar trend analysis was conducted for seasonal rainfall (results not shown).

Figure S1 shows the values of the “Z-score” for Tx, Tn, and Tmean for each of the 12 seasons. Horizontal lines on the plot show the values required for significance at various confidence levels. Only two seasons were identified in which none of these three variables were statistically significant (at p <0.1): DJF and JFM. The only temperature variable that did not have a positive Z-score was Tn for the FMA season. The only season with a statistically significant (p < 0.1), and downward, trend in rainfall (not shown) was MAM.

**Added utility from the availability of daily data**

The availability of daily meteorological data (versus monthly, seasonal, or annual) allows for much more detailed analyses of environmental fluctuations and their possible connection to malaria. Data with high temporal resolution are highly desired, for example, if there are substantial nonlinearities in the system being modeled [1]. Rainfall can have differential effects on maximum versus minimum temperatures and since the incidence, sequence and intensity of daily rainfall events can vary substantially (even between months having roughly the same total precipitation), rainfall-temperature relationships are likely to exhibit potentially important sub-monthly variations as well.

One example of an analysis that can be conducted on daily data is examination of long-term temporal trends in the maximum or minimum values of Tx or Tn that occur *within* a given month. The adjusted temperature data sets were used for this purpose, first to generate four new time series of the maximum and minimum values occurring in each month of Tx and Tn. The Mann-Kendall test was then applied to each time series (1979-2009) separately for each calendar month. The results for the case of maximum daily values of Tx and Tn are shown in Figure S2, which is presented in a format similar to Figure 1. Several months of the year show statistically significant trends in this metric, more so for Tn than Tx. The months of December, January and February had fewer statistically significant trends than other months, and for the months of July, August and September both Tx and Tn had significant trends simultaneously.

Figure S3 shows and example of how the behavior of Tx and Tn can vary substantially during the same calendar month. Plotted are the departures of daily Tx and Tn from the long-term (1979-2009) monthly average for April 1993. The 30 observations for April are ranked from smallest to largest in the figure. Tx values clearly exhibit many more positive departures than Tn. Indeed, Tx during the month of April 1993 was 1.4 deg. C above the long-term average, while the monthly departure from average for Tn was -1.6 deg. C. It is noted that rainfall in April 1993 was only 74% of the 1980-2009 median value for April. As described in the main text (Figure 6), unusually dry conditions tend to have the effect of increasing Tx while Tn values decrease.

As mentioned previously, the daily sequence of rainfall can be an important factor in the control of Tx and Tn. Plotted in Figure S4 are the daily departures of Tx and Tn from their respective monthly average values (to avoid any possible influence of a long-term trend in the analysis) during two months when total monthly rainfall differed by < 5% (these were April 1996 and April 1999). The daily departures were again ranked from smallest to largest in the figure. When comparing the daily ranked departures Figure S4 indicates that values of Tn in 1999 tended to exceed (i.e., be greater in absolute value) those in 1996. A similar tendency is seen for the case of Tx values. For this to hold, the variance in both daily Tx and Tn had to be larger in April 1999 than in April 1996. Indeed, the variance of daily Tx values was found to be 18% higher in 1999 than in 1996; for Tn it was 39% higher in 1999 than in 1996. In this example, even for two months with nearly identical total monthly rainfall, there were substantial differences in daily temperature variance characteristics.

In a final, but by no means exhaustive example, daily rainfall events for April 1996 and April 1999 were examined. As mentioned, the total rainfall for these two months varied by < 5% (213 mm in April 1999 and 222 mm in April 1996). What is plotted in Figure S5 is the ratio of ranked daily rainfall to the daily average rainfall for each month. The figure indicates that in April 1996 there was a larger contribution to the monthly total rainfall from heavier rainfall events than in April 1999. A comparison of the variance in daily rainfall for the two months indicates that it was over 30% higher in 1996 than in 1999 – just the opposite as for temperature where the variance of both Tx and Tn was greater in 1999 than 1996.

All of the above examples indicate the complexity that exists in the relationships between rainfall and temperature at the Kericho location and highlight the need for high quality, daily meteorological data. Such data is clearly also needed to model the extent to which these variations in the physical climate may impact the environmental suitability of malaria.

**Reference**

[1] Alonso D, Bouma MJ, Pascual M: **Epidemic malaria and warmer temperatures in recent decades in an East African highland**. *Proceedings of the Royal Society B* 2010.


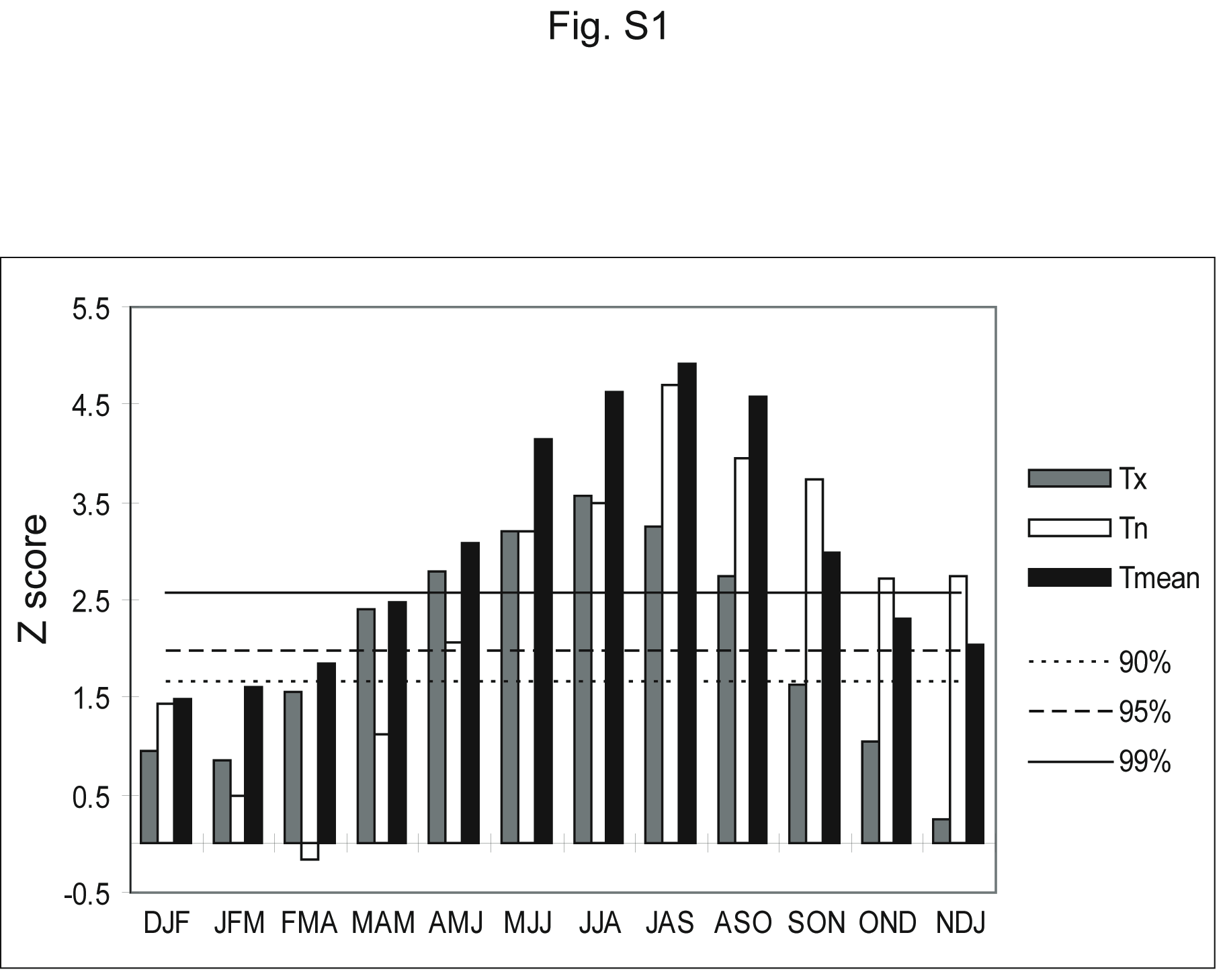


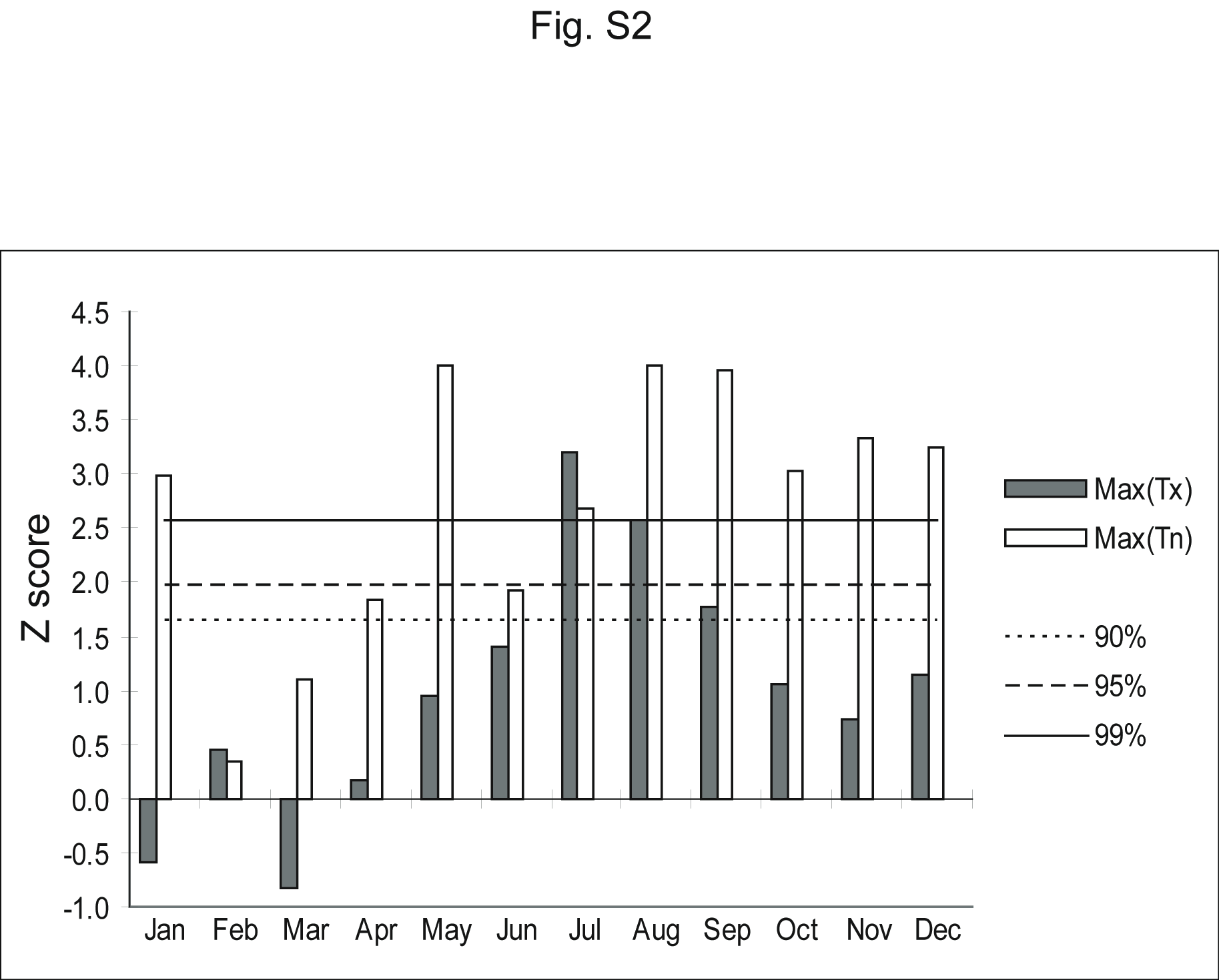


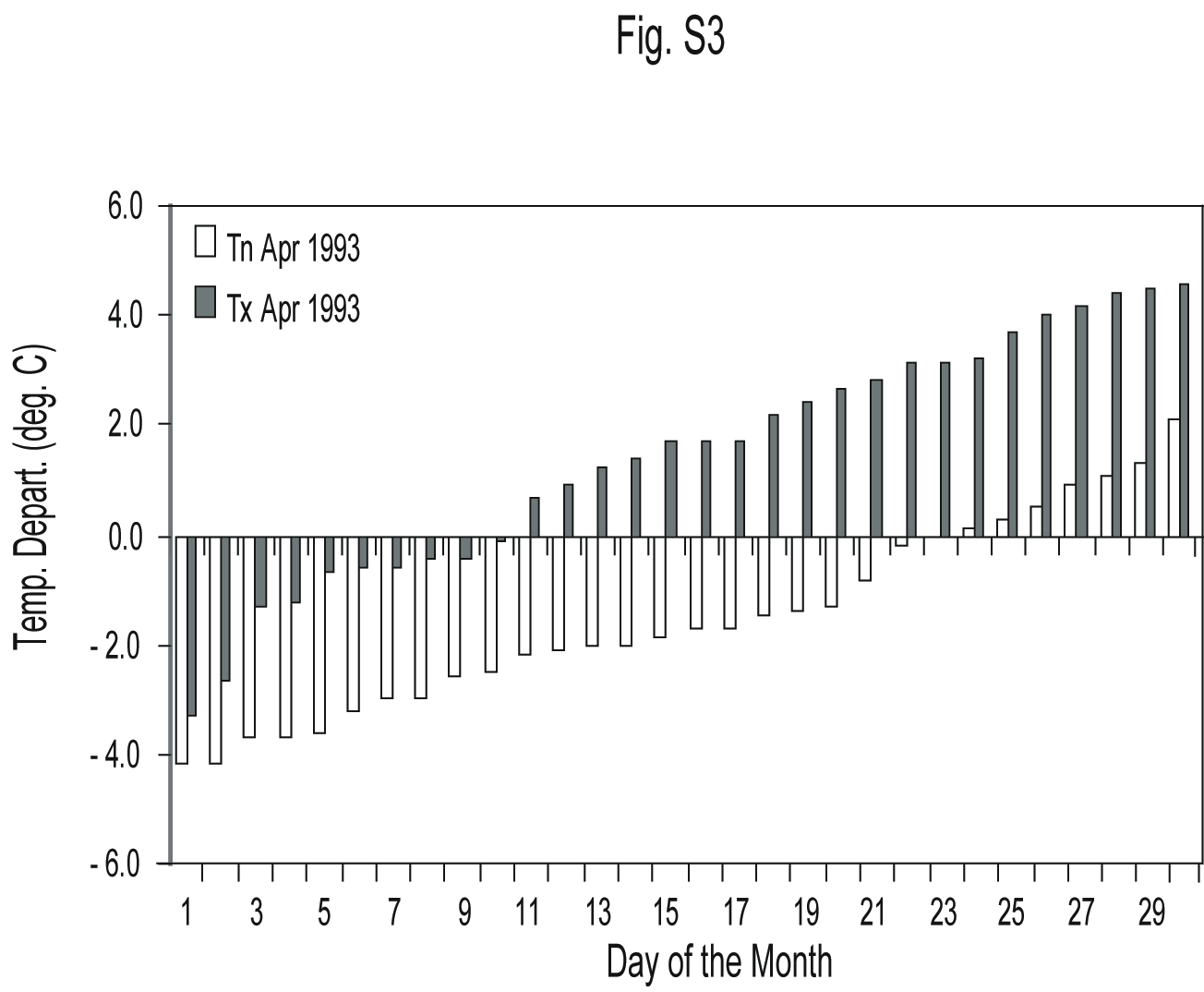


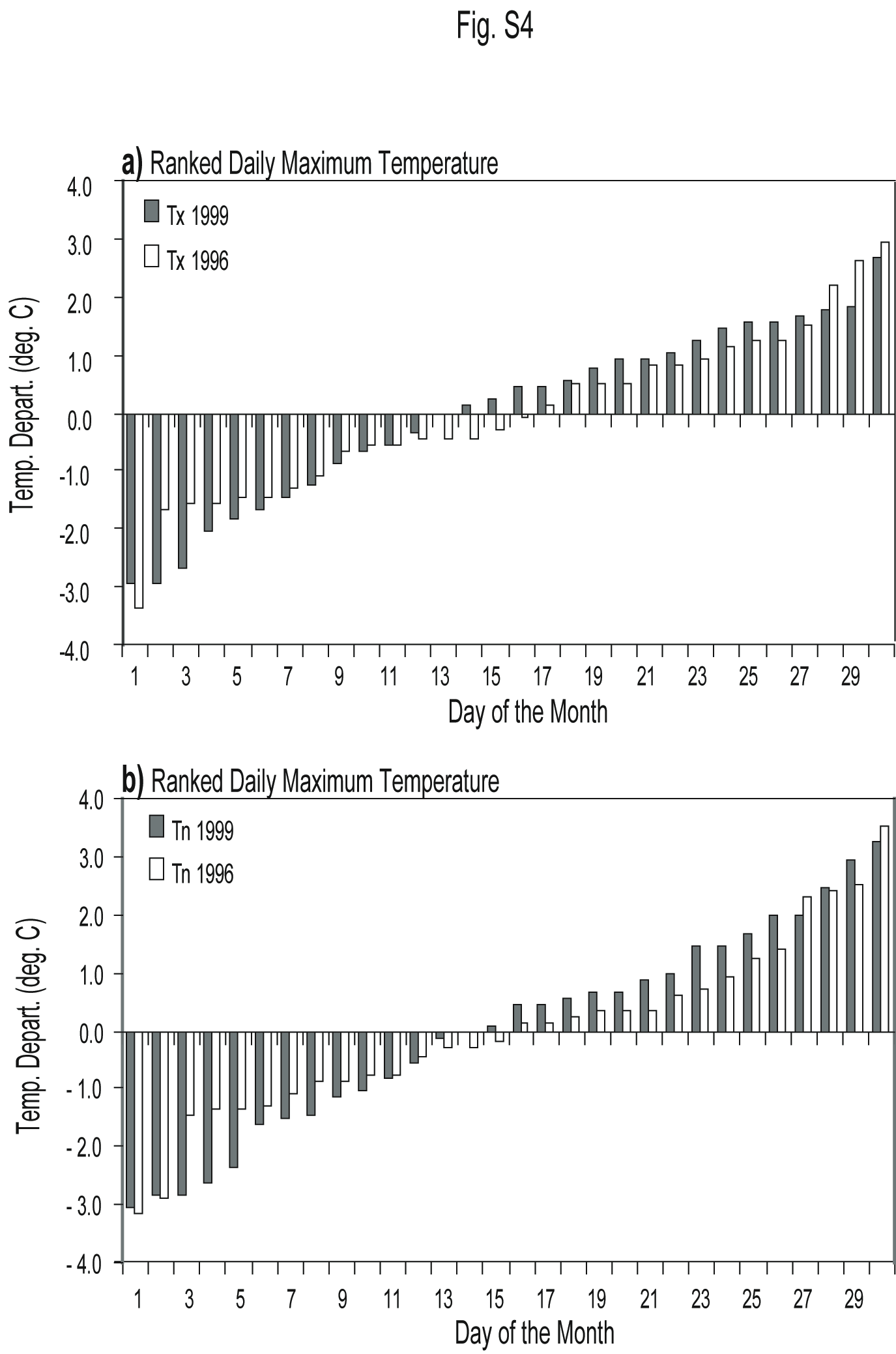


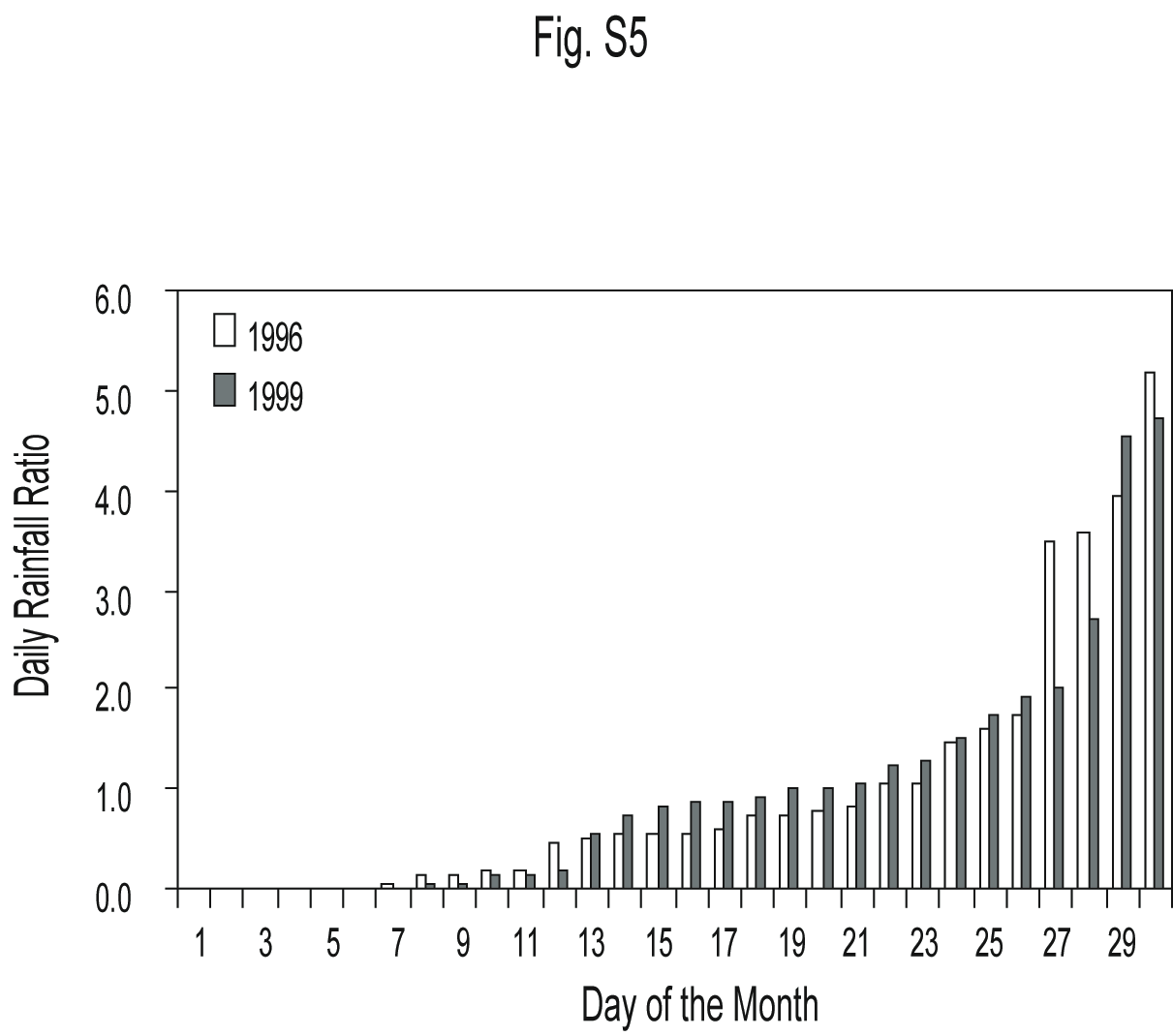


**APPENDIX – Monthly Tx, Tn, Tmean and Rainfall at the Kericho Station**

The monthly temperatures listed below are the adjusted values as described in the main text, they are all given in deg. C. Average monthly rainfall is given in mm/day. Missing values for all variables are identified as -999. These data are being provided with the explicit consent of the Kenyan Meteorological Department.

| **Year** | **Month** | **Tx** | **Tn** | **Tmean** | **Rainfall** |
| --- | --- | --- | --- | --- | --- |
| 1979 | 1 | 24.0 | 11.8 | 17.9 | -999 |
| 1979 | 2 | 23.5 | 11.5 | 17.5 | -999 |
| 1979 | 3 | 25.1 | 10.9 | 18.0 | -999 |
| 1979 | 4 | 23.6 | 12.2 | 17.9 | -999 |
| 1979 | 5 | 22.9 | 10.9 | 16.9 | -999 |
| 1979 | 6 | 22.1 | 11.4 | 16.8 | -999 |
| 1979 | 7 | 22.1 | 10.2 | 16.2 | -999 |
| 1979 | 8 | 23.0 | 10.1 | 16.5 | -999 |
| 1979 | 9 | 23.9 | 10.2 | 17.1 | -999 |
| 1979 | 10 | 25.2 | 11.1 | 18.2 | -999 |
| 1979 | 11 | -999 | -999 | -999 | -999 |
| 1979 | 12 | 24.8 | 10.3 | 17.5 | -999 |
| 1980 | 1 | 25.9 | 10.2 | 18.1 | 3.1 |
| 1980 | 2 | 27.0 | 11.1 | 19.0 | 1.2 |
| 1980 | 3 | 26.1 | 11.3 | 18.7 | 3.2 |
| 1980 | 4 | 24.8 | 11.0 | 17.9 | 7.5 |
| 1980 | 5 | 23.1 | 11.6 | 17.4 | 10.3 |
| 1980 | 6 | 23.0 | 10.7 | 16.8 | 7.1 |
| 1980 | 7 | 22.3 | 10.1 | 16.2 | 6.0 |
| 1980 | 8 | 22.7 | 10.2 | 16.4 | 6.5 |
| 1980 | 9 | 24.2 | 9.5 | 16.9 | 4.8 |
| 1980 | 10 | 24.4 | 10.2 | 17.3 | 3.4 |
| 1980 | 11 | 23.4 | 11.3 | 17.3 | 3.4 |
| 1980 | 12 | 24.5 | 9.9 | 17.2 | 1.1 |
| 1981 | 1 | 26.5 | 10.6 | 18.5 | 0.6 |
| 1981 | 2 | 26.5 | 11.0 | 18.7 | 2.7 |
| 1981 | 3 | -999 | -999 | -999 | 12.0 |
| 1981 | 4 | 23.3 | 11.5 | 17.4 | 9.8 |
| 1981 | 5 | 23.2 | 10.3 | 16.8 | 6.9 |
| 1981 | 6 | 23.4 | 10.0 | 16.7 | 3.1 |
| 1981 | 7 | 21.3 | 10.0 | 15.7 | 7.7 |
| 1981 | 8 | 22.3 | 10.0 | 16.2 | 7.9 |
| 1981 | 9 | 22.8 | 9.9 | 16.4 | 8.2 |
| 1981 | 10 | 23.9 | 10.6 | 17.3 | 4.4 |
| 1981 | 11 | 23.6 | 10.8 | 17.2 | 4.0 |
| 1981 | 12 | 24.6 | 10.5 | 17.6 | 2.1 |
| 1982 | 1 | 26.0 | 10.5 | 18.3 | 0.9 |
| 1982 | 2 | 27.7 | 10.3 | 19.0 | 2.4 |
| 1982 | 3 | 23.7 | 11.5 | 17.6 | 4.3 |
| 1982 | 4 | 22.7 | 11.4 | 17.0 | 8.9 |
| 1982 | 5 | 23.1 | 10.6 | 16.9 | 14.8 |
| 1982 | 6 | 22.3 | 10.4 | 16.4 | 3.3 |
| 1982 | 7 | 22.4 | 10.4 | 16.4 | 6.2 |
| 1982 | 8 | 22.4 | 10.4 | 16.4 | 10.4 |
| 1982 | 9 | 24.1 | 10.1 | 17.1 | 5.1 |
| 1982 | 10 | 22.8 | 10.4 | 16.6 | 6.1 |
| 1982 | 11 | -999 | -999 | -999 | 13.0 |
| 1982 | 12 | 24.0 | 10.9 | 17.5 | 4.9 |
| 1983 | 1 | 25.1 | 10.1 | 17.6 | 2.0 |
| 1983 | 2 | 26.0 | 11.8 | 18.9 | 1.5 |
| 1983 | 3 | 27.2 | 11.8 | 19.5 | 2.1 |
| 1983 | 4 | 24.6 | 11.4 | 18.0 | 8.0 |
| 1983 | 5 | 23.7 | 11.0 | 17.3 | 6.9 |
| 1983 | 6 | 23.1 | 11.2 | 17.1 | 12.3 |
| 1983 | 7 | 22.5 | 11.1 | 16.8 | 7.3 |
| 1983 | 8 | 22.3 | 10.7 | 16.5 | 7.9 |
| 1983 | 9 | 23.3 | 10.0 | 16.7 | 9.1 |
| 1983 | 10 | 23.0 | 11.0 | 17.0 | 7.1 |
| 1983 | 11 | 23.3 | 10.8 | 17.0 | 4.5 |
| 1983 | 12 | 23.0 | 11.0 | 17.0 | 2.5 |
| 1984 | 1 | 24.3 | 9.8 | 17.1 | 1.8 |
| 1984 | 2 | 26.7 | 9.7 | 18.2 | 1.2 |
| 1984 | 3 | 27.4 | 11.9 | 19.6 | 1.6 |
| 1984 | 4 | 25.2 | 11.3 | 18.3 | 8.6 |
| 1984 | 5 | 23.9 | 10.6 | 17.3 | 5.0 |
| 1984 | 6 | 22.7 | 10.3 | 16.5 | 5.1 |
| 1984 | 7 | 22.1 | 10.4 | 16.3 | 6.5 |
| 1984 | 8 | 22.8 | 10.7 | 16.7 | 5.7 |
| 1984 | 9 | 23.8 | 9.9 | 16.8 | 3.6 |
| 1984 | 10 | 23.7 | 11.2 | 17.4 | 3.6 |
| 1984 | 11 | 23.0 | 10.9 | 16.9 | 4.8 |
| 1984 | 12 | 23.8 | 10.7 | 17.2 | 1.5 |
| 1985 | 1 | 25.7 | 11.2 | 18.4 | 2.4 |
| 1985 | 2 | 23.6 | 10.7 | 17.2 | 4.6 |
| 1985 | 3 | 25.1 | 12.1 | 18.6 | 5.4 |
| 1985 | 4 | 22.4 | 11.7 | 17.0 | 13.0 |
| 1985 | 5 | 22.5 | 11.6 | 17.1 | 10.2 |
| 1985 | 6 | 22.4 | 11.2 | 16.8 | 5.2 |
| 1985 | 7 | 21.5 | 9.9 | 15.7 | 9.7 |
| 1985 | 8 | 21.9 | 10.6 | 16.2 | 7.3 |
| 1985 | 9 | 23.5 | 10.5 | 17.0 | 8.1 |
| 1985 | 10 | 24.6 | 11.3 | 17.9 | 1.8 |
| 1985 | 11 | 23.6 | 11.6 | 17.6 | 4.2 |
| 1985 | 12 | 24.8 | 10.8 | 17.8 | 2.1 |
| 1986 | 1 | 25.1 | 10.0 | 17.5 | 1.3 |
| 1986 | 2 | 26.8 | 10.3 | 18.6 | 1.6 |
| 1986 | 3 | 26.0 | 10.4 | 18.2 | 3.8 |
| 1986 | 4 | 23.5 | 12.0 | 17.7 | 12.0 |
| 1986 | 5 | 23.1 | 11.2 | 17.1 | 10.0 |
| 1986 | 6 | 21.7 | 11.4 | 16.5 | 3.0 |
| 1986 | 7 | 22.0 | 10.3 | 16.2 | 4.5 |
| 1986 | 8 | 23.1 | 10.0 | 16.5 | 4.6 |
| 1986 | 9 | 23.6 | 10.1 | 16.8 | 6.5 |
| 1986 | 10 | 24.4 | 11.2 | 17.8 | 2.2 |
| 1986 | 11 | 23.8 | 11.4 | 17.6 | 2.1 |
| 1986 | 12 | 24.0 | 11.0 | 17.5 | 1.7 |
| 1987 | 1 | 25.4 | 11.2 | 18.3 | 3.5 |
| 1987 | 2 | 26.2 | 11.0 | 18.6 | 5.3 |
| 1987 | 3 | 25.4 | 11.5 | 18.5 | 7.9 |
| 1987 | 4 | 24.5 | 11.8 | 18.1 | 6.8 |
| 1987 | 5 | 22.9 | 11.7 | 17.3 | 7.3 |
| 1987 | 6 | 22.5 | 11.9 | 17.2 | 7.3 |
| 1987 | 7 | 23.8 | 10.5 | 17.2 | 4.1 |
| 1987 | 8 | 23.5 | 11.3 | 17.4 | 5.8 |
| 1987 | 9 | 25.1 | 10.8 | 18.0 | 4.2 |
| 1987 | 10 | 24.7 | 10.6 | 17.7 | 4.1 |
| 1987 | 11 | 23.5 | 11.8 | 17.6 | 6.9 |
| 1987 | 12 | 25.4 | 10.2 | 17.8 | 2.3 |
| 1988 | 1 | 24.4 | 10.8 | 17.6 | 11.7 |
| 1988 | 2 | 25.8 | 11.7 | 18.7 | 2.6 |
| 1988 | 3 | 24.8 | 11.6 | 18.2 | 6.0 |
| 1988 | 4 | 23.4 | 12.5 | 18.0 | 8.9 |
| 1988 | 5 | 23.8 | 11.3 | 17.5 | 5.9 |
| 1988 | 6 | 22.9 | 10.6 | 16.8 | 4.9 |
| 1988 | 7 | 22.0 | 10.9 | 16.4 | 5.2 |
| 1988 | 8 | 22.2 | 10.8 | 16.5 | 8.9 |
| 1988 | 9 | 22.9 | 10.9 | 16.9 | 7.3 |
| 1988 | 10 | 23.7 | 9.9 | 16.8 | 6.9 |
| 1988 | 11 | 23.3 | 10.5 | 16.9 | 4.5 |
| 1988 | 12 | 24.1 | 10.7 | 17.4 | 1.2 |
| 1989 | 1 | 23.9 | 10.6 | 17.3 | 1.9 |
| 1989 | 2 | 25.1 | 10.6 | 17.9 | 4.2 |
| 1989 | 3 | 24.5 | 10.3 | 17.4 | 14.2 |
| 1989 | 4 | 23.2 | 11.1 | 17.2 | 6.4 |
| 1989 | 5 | 22.7 | 10.5 | 16.6 | 7.4 |
| 1989 | 6 | 22.9 | 10.6 | 16.7 | 1.8 |
| 1989 | 7 | 21.9 | 11.0 | 16.4 | -999 |
| 1989 | 8 | 22.6 | 10.9 | 16.7 | 5.8 |
| 1989 | 9 | 23.5 | 10.7 | 17.1 | 6.5 |
| 1989 | 10 | 23.3 | 10.6 | 17.0 | 5.2 |
| 1989 | 11 | 23.7 | 10.9 | 17.3 | 3.1 |
| 1989 | 12 | 23.9 | 10.9 | 17.4 | 5.0 |
| 1990 | 1 | 24.9 | 10.3 | 17.6 | 3.6 |
| 1990 | 2 | 24.7 | 11.9 | 18.3 | 7.5 |
| 1990 | 3 | 24.0 | 11.7 | 17.9 | 7.5 |
| 1990 | 4 | 23.4 | 12.1 | 17.7 | 8.3 |
| 1990 | 5 | 23.8 | 11.7 | 17.7 | 5.1 |
| 1990 | 6 | 23.3 | 10.5 | 16.9 | 3.0 |
| 1990 | 7 | 23.1 | 10.8 | 16.9 | 3.6 |
| 1990 | 8 | 22.9 | 10.6 | 16.8 | 6.6 |
| 1990 | 9 | 24.2 | 10.1 | 17.1 | 5.3 |
| 1990 | 10 | 24.3 | 10.4 | 17.4 | 4.6 |
| 1990 | 11 | 24.1 | 10.1 | 17.1 | 3.9 |
| 1990 | 12 | 24.3 | 10.1 | 17.2 | 3.0 |
| 1991 | 1 | 25.0 | 10.4 | 17.7 | 6.7 |
| 1991 | 2 | 26.3 | 10.2 | 18.3 | -999 |
| 1991 | 3 | 26.0 | 11.1 | 18.6 | 6.1 |
| 1991 | 4 | 24.2 | 11.3 | 17.8 | 4.0 |
| 1991 | 5 | 22.9 | 12.0 | 17.4 | 11.9 |
| 1991 | 6 | 23.3 | 12.2 | 17.8 | -999 |
| 1991 | 7 | 21.8 | 11.2 | 16.5 | 3.8 |
| 1991 | 8 | 22.9 | 11.3 | 17.1 | 6.4 |
| 1991 | 9 | 24.1 | 10.2 | 17.2 | 5.5 |
| 1991 | 10 | 23.5 | 10.3 | 16.9 | 7.3 |
| 1991 | 11 | 23.7 | 10.1 | 16.9 | 4.1 |
| 1991 | 12 | 24.2 | 10.5 | 17.3 | 1.2 |
| 1992 | 1 | 26.6 | 10.5 | 18.6 | 0.9 |
| 1992 | 2 | 25.9 | 11.4 | 18.6 | 4.3 |
| 1992 | 3 | 27.4 | 11.2 | 19.3 | 4.9 |
| 1992 | 4 | 24.5 | 11.7 | 18.1 | 7.1 |
| 1992 | 5 | 23.7 | 11.0 | 17.4 | 6.8 |
| 1992 | 6 | 22.9 | 12.0 | 17.4 | 7.2 |
| 1992 | 7 | 22.1 | 10.6 | 16.4 | 6.1 |
| 1992 | 8 | 22.5 | 11.5 | 17.0 | 7.4 |
| 1992 | 9 | 23.6 | 10.8 | 17.2 | 5.7 |
| 1992 | 10 | 23.2 | 11.2 | 17.2 | 8.4 |
| 1992 | 11 | 23.9 | 9.8 | 16.8 | 3.0 |
| 1992 | 12 | 23.7 | 11.1 | 17.4 | 3.3 |
| 1993 | 1 | 22.8 | 11.9 | 17.3 | 4.7 |
| 1993 | 2 | 24.2 | 11.0 | 17.6 | 4.1 |
| 1993 | 3 | 26.4 | 9.6 | 18.0 | 1.3 |
| 1993 | 4 | 25.6 | 10.1 | 17.8 | 5.9 |
| 1993 | 5 | 23.3 | 11.3 | 17.3 | 9.1 |
| 1993 | 6 | 22.5 | 11.3 | 16.9 | 6.9 |
| 1993 | 7 | 22.6 | 10.6 | 16.6 | 4.9 |
| 1993 | 8 | 23.5 | 10.2 | 16.9 | 3.6 |
| 1993 | 9 | 24.4 | 10.5 | 17.4 | 3.6 |
| 1993 | 10 | 24.9 | 10.1 | 17.5 | 5.3 |
| 1993 | 11 | 24.6 | 11.2 | 17.9 | 2.3 |
| 1993 | 12 | 25.1 | 11.2 | 18.2 | 3.3 |
| 1994 | 1 | 26.5 | 10.6 | 18.6 | 1.3 |
| 1994 | 2 | 26.6 | 9.9 | 18.3 | 1.8 |
| 1994 | 3 | 25.6 | 11.2 | 18.4 | 6.7 |
| 1994 | 4 | 24.8 | 11.4 | 18.1 | 7.5 |
| 1994 | 5 | 23.2 | 11.0 | 17.1 | 12.7 |
| 1994 | 6 | 22.5 | 11.9 | 17.2 | 4.6 |
| 1994 | 7 | 22.1 | 11.2 | 16.6 | 8.0 |
| 1994 | 8 | 22.8 | 11.0 | 16.9 | 6.0 |
| 1994 | 9 | 24.7 | 10.0 | 17.3 | 3.3 |
| 1994 | 10 | 24.3 | 10.2 | 17.3 | 3.8 |
| 1994 | 11 | 22.6 | 11.9 | 17.2 | 7.7 |
| 1994 | 12 | 24.4 | 10.1 | 17.3 | 1.6 |
| 1995 | 1 | 26.4 | 11.2 | 18.8 | 1.0 |
| 1995 | 2 | 25.8 | 10.3 | 18.0 | 3.3 |
| 1995 | 3 | 25.1 | 10.8 | 18.0 | 6.7 |
| 1995 | 4 | 24.4 | 11.8 | 18.1 | 7.2 |
| 1995 | 5 | 23.6 | 11.3 | 17.4 | 7.7 |
| 1995 | 6 | 23.7 | 12.2 | 18.0 | 6.9 |
| 1995 | 7 | 22.2 | 11.3 | 16.8 | 2.6 |
| 1995 | 8 | 23.6 | 11.1 | 17.4 | 4.3 |
| 1995 | 9 | 24.0 | 11.4 | 17.7 | 6.1 |
| 1995 | 10 | 23.6 | 11.3 | 17.5 | 9.3 |
| 1995 | 11 | 23.7 | 11.7 | 17.7 | 5.8 |
| 1995 | 12 | 24.2 | 9.4 | 16.8 | 3.0 |
| 1996 | 1 | 25.1 | 9.5 | 17.3 | 2.2 |
| 1996 | 2 | 24.9 | 10.8 | 17.8 | 6.7 |
| 1996 | 3 | 24.8 | 11.3 | 18.1 | 5.0 |
| 1996 | 4 | 24.0 | 10.7 | 17.3 | 7.4 |
| 1996 | 5 | 23.7 | 11.5 | 17.6 | 6.7 |
| 1996 | 6 | 22.3 | 12.0 | 17.2 | 2.3 |
| 1996 | 7 | 22.4 | 11.0 | 16.7 | 8.9 |
| 1996 | 8 | 23.2 | 11.0 | 17.1 | 5.6 |
| 1996 | 9 | 23.9 | 10.9 | 17.4 | 5.4 |
| 1996 | 10 | 24.3 | 10.6 | 17.5 | 7.8 |
| 1996 | 11 | 23.6 | 11.0 | 17.3 | 5.8 |
| 1996 | 12 | 24.6 | 11.4 | 18.0 | 2.4 |
| 1997 | 1 | 25.8 | 10.4 | 18.1 | 1.5 |
| 1997 | 2 | 28.3 | 9.1 | 18.7 | -999 |
| 1997 | 3 | 28.7 | 10.9 | 19.8 | 2.1 |
| 1997 | 4 | 23.5 | 12.2 | 17.8 | 10.0 |
| 1997 | 5 | 23.6 | 10.6 | 17.1 | 2.4 |
| 1997 | 6 | 23.6 | 11.1 | 17.3 | 4.3 |
| 1997 | 7 | 22.7 | 11.3 | 17.0 | 5.9 |
| 1997 | 8 | 23.9 | 10.9 | 17.4 | 3.4 |
| 1997 | 9 | 26.8 | 9.1 | 18.0 | 1.2 |
| 1997 | 10 | 24.4 | 11.2 | 17.8 | 6.7 |
| 1997 | 11 | 22.5 | 12.0 | 17.2 | 12.6 |
| 1997 | 12 | -999 | 12.3 | -999 | 7.1 |
| 1998 | 1 | -999 | 12.4 | -999 | 10.5 |
| 1998 | 2 | -999 | 12.1 | -999 | 3.7 |
| 1998 | 3 | -999 | 12.1 | -999 | 2.0 |
| 1998 | 4 | 25.6 | 13.2 | 19.4 | 8.2 |
| 1998 | 5 | 24.2 | 13.3 | 18.8 | 8.4 |
| 1998 | 6 | 23.4 | 11.2 | 17.3 | 6.4 |
| 1998 | 7 | 22.7 | 11.6 | 17.1 | 6.6 |
| 1998 | 8 | 23.7 | 11.7 | 17.7 | 5.4 |
| 1998 | 9 | 24.3 | 11.2 | 17.7 | 4.7 |
| 1998 | 10 | 24.0 | 11.6 | 17.8 | 10.5 |
| 1998 | 11 | 24.0 | 11.1 | 17.5 | 3.5 |
| 1998 | 12 | 25.8 | 9.9 | 17.8 | 0.6 |
| 1999 | 1 | 25.6 | 10.2 | 17.9 | 4.5 |
| 1999 | 2 | 28.0 | 10.6 | 19.3 | 1.0 |
| 1999 | 3 | 24.4 | 12.0 | 18.2 | 9.6 |
| 1999 | 4 | 24.1 | 10.5 | 17.3 | 7.0 |
| 1999 | 5 | 23.4 | 10.7 | 17.0 | 8.4 |
| 1999 | 6 | 23.6 | 10.0 | 16.8 | 3.8 |
| 1999 | 7 | 22.4 | 10.3 | 16.4 | 3.3 |
| 1999 | 8 | 22.9 | 10.9 | 16.9 | 5.6 |
| 1999 | 9 | 24.0 | 10.5 | 17.3 | 4.9 |
| 1999 | 10 | 23.4 | 11.1 | 17.2 | 6.9 |
| 1999 | 11 | 24.0 | 11.1 | 17.5 | 2.7 |
| 1999 | 12 | 24.1 | 10.2 | 17.1 | 2.2 |
| 2000 | 1 | 26.5 | 9.9 | 18.2 | 2.0 |
| 2000 | 2 | 27.8 | 9.5 | 18.6 | 1.1 |
| 2000 | 3 | 26.8 | 10.8 | 18.8 | 3.1 |
| 2000 | 4 | 25.1 | 11.6 | 18.3 | 5.4 |
| 2000 | 5 | 24.1 | 11.3 | 17.7 | 7.1 |
| 2000 | 6 | 23.4 | 11.3 | 17.3 | 5.7 |
| 2000 | 7 | 22.7 | 11.8 | 17.2 | 5.1 |
| 2000 | 8 | 23.1 | 10.8 | 17.0 | 5.9 |
| 2000 | 9 | 24.4 | 10.9 | 17.6 | 5.4 |
| 2000 | 10 | 24.0 | 10.6 | 17.3 | 5.0 |
| 2000 | 11 | 23.8 | 11.2 | 17.5 | 5.8 |
| 2000 | 12 | 24.1 | 11.1 | 17.6 | 6.5 |
| 2001 | 1 | 23.0 | 11.9 | 17.4 | 14.4 |
| 2001 | 2 | 26.1 | 10.2 | 18.2 | 2.5 |
| 2001 | 3 | 25.6 | 10.8 | 18.2 | 4.3 |
| 2001 | 4 | 23.9 | 11.6 | 17.7 | 7.7 |
| 2001 | 5 | 23.9 | 12.2 | 18.1 | 10.4 |
| 2001 | 6 | 22.5 | 11.1 | 16.8 | 7.9 |
| 2001 | 7 | 22.5 | 10.8 | 16.7 | 4.1 |
| 2001 | 8 | 23.4 | 11.2 | 17.3 | 3.7 |
| 2001 | 9 | 23.9 | 11.0 | 17.5 | 6.0 |
| 2001 | 10 | 24.6 | 11.3 | 17.9 | 9.6 |
| 2001 | 11 | 23.2 | 11.6 | 17.4 | 7.8 |
| 2001 | 12 | 25.5 | 11.0 | 18.3 | 1.9 |
| 2002 | 1 | 24.8 | 11.3 | 18.0 | 5.0 |
| 2002 | 2 | 26.9 | 11.3 | 19.1 | 1.7 |
| 2002 | 3 | 25.6 | 10.5 | 18.0 | 4.8 |
| 2002 | 4 | 24.9 | 11.4 | 18.2 | 8.2 |
| 2002 | 5 | 24.0 | 11.9 | 17.9 | 11.6 |
| 2002 | 6 | 23.4 | 11.2 | 17.3 | 4.1 |
| 2002 | 7 | 24.1 | 10.2 | 17.1 | 4.7 |
| 2002 | 8 | 23.2 | 11.0 | 17.1 | 7.6 |
| 2002 | 9 | 24.9 | 10.0 | 17.4 | 2.2 |
| 2002 | 10 | 24.4 | 10.9 | 17.6 | 4.3 |
| 2002 | 11 | 24.0 | 11.3 | 17.7 | 6.1 |
| 2002 | 12 | 23.8 | 12.0 | 17.9 | 7.4 |
| 2003 | 1 | 25.3 | 10.2 | 17.7 | 0.5 |
| 2003 | 2 | 27.6 | 11.1 | 19.4 | 0.8 |
| 2003 | 3 | 27.6 | 12.0 | 19.8 | 6.2 |
| 2003 | 4 | 24.7 | 12.6 | 18.7 | 10.8 |
| 2003 | 5 | 23.5 | 12.1 | 17.8 | 11.0 |
| 2003 | 6 | 23.1 | 12.0 | 17.5 | 4.9 |
| 2003 | 7 | 22.6 | 11.5 | 17.1 | 4.2 |
| 2003 | 8 | 23.1 | 11.9 | 17.5 | 6.4 |
| 2003 | 9 | 24.3 | 10.5 | 17.4 | 6.4 |
| 2003 | 10 | 24.5 | 10.9 | 17.7 | 6.0 |
| 2003 | 11 | 24.2 | 11.7 | 18.0 | 3.6 |
| 2003 | 12 | 24.6 | 9.9 | 17.3 | 2.0 |
| 2004 | 1 | 25.3 | 11.4 | 18.4 | 4.2 |
| 2004 | 2 | 25.8 | 12.3 | 19.0 | 3.0 |
| 2004 | 3 | 26.1 | 11.9 | 19.0 | 5.3 |
| 2004 | 4 | 23.6 | 12.4 | 18.0 | 9.3 |
| 2004 | 5 | 24.3 | 11.6 | 17.9 | 5.2 |
| 2004 | 6 | 23.1 | 11.6 | 17.4 | 4.0 |
| 2004 | 7 | 23.3 | 10.4 | 16.9 | 3.3 |
| 2004 | 8 | 23.4 | 11.6 | 17.5 | 5.4 |
| 2004 | 9 | 24.0 | 11.4 | 17.7 | 6.1 |
| 2004 | 10 | 24.1 | 10.8 | 17.5 | 1.8 |
| 2004 | 11 | 23.3 | 11.1 | 17.2 | 4.9 |
| 2004 | 12 | 24.5 | 11.3 | 17.9 | -999 |
| 2005 | 1 | 25.5 | 11.7 | 18.6 | 4.8 |
| 2005 | 2 | 27.6 | 12.0 | 19.8 | 2.2 |
| 2005 | 3 | 26.0 | 12.0 | 19.0 | 7.8 |
| 2005 | 4 | 25.3 | 12.1 | 18.7 | 5.8 |
| 2005 | 5 | 23.0 | 12.6 | 17.8 | 8.4 |
| 2005 | 6 | 23.4 | 12.0 | 17.7 | 6.8 |
| 2005 | 7 | 22.4 | 11.6 | 17.0 | 6.2 |
| 2005 | 8 | 23.3 | 10.9 | 17.1 | 7.0 |
| 2005 | 9 | 24.1 | 11.6 | 17.8 | 3.2 |
| 2005 | 10 | 24.6 | 11.2 | 17.9 | 3.3 |
| 2005 | 11 | 24.6 | 11.0 | 17.8 | 2.8 |
| 2005 | 12 | 26.8 | 11.3 | 19.1 | 0.4 |
| 2006 | 1 | 26.6 | 12.0 | 19.3 | 2.5 |
| 2006 | 2 | 27.7 | 12.4 | 20.0 | 2.1 |
| 2006 | 3 | 24.5 | 11.9 | 18.2 | 7.2 |
| 2006 | 4 | 23.6 | 12.0 | 17.8 | 11.9 |
| 2006 | 5 | 23.3 | 11.5 | 17.4 | 8.1 |
| 2006 | 6 | 23.1 | 11.3 | 17.2 | 5.5 |
| 2006 | 7 | 22.8 | 11.7 | 17.3 | 7.2 |
| 2006 | 8 | 23.5 | 11.3 | 17.4 | 5.0 |
| 2006 | 9 | 24.2 | 10.9 | 17.5 | 4.3 |
| 2006 | 10 | 24.8 | 10.4 | 17.6 | 5.4 |
| 2006 | 11 | 22.4 | 12.0 | 17.2 | 10.9 |
| 2006 | 12 | 22.7 | 12.4 | 17.6 | 10.7 |
| 2007 | 1 | 24.5 | 11.4 | 17.9 | 4.9 |
| 2007 | 2 | 24.7 | 11.8 | 18.3 | 4.6 |
| 2007 | 3 | 25.8 | 10.5 | 18.1 | 4.0 |
| 2007 | 4 | -999 | 11.8 | -999 | 7.4 |
| 2007 | 5 | -999 | 11.7 | -999 | 7.3 |
| 2007 | 6 | -999 | 12.3 | -999 | 6.9 |
| 2007 | 7 | -999 | 11.4 | -999 | 5.7 |
| 2007 | 8 | -999 | 11.3 | -999 | 7.5 |
| 2007 | 9 | -999 | 11.2 | -999 | 10.8 |
| 2007 | 10 | 23.9 | 10.7 | 17.3 | 4.1 |
| 2007 | 11 | 24.3 | 11.0 | 17.6 | 1.9 |
| 2007 | 12 | 25.1 | 10.4 | 17.8 | 1.7 |
| 2008 | 1 | 25.8 | 10.6 | 18.2 | 1.4 |
| 2008 | 2 | 26.2 | 10.5 | 18.3 | 2.2 |
| 2008 | 3 | 25.8 | 11.2 | 18.5 | 9.5 |
| 2008 | 4 | 24.2 | 10.9 | 17.5 | 6.3 |
| 2008 | 5 | 23.6 | 10.7 | 17.1 | 6.7 |
| 2008 | 6 | 22.9 | 11.5 | 17.2 | 4.4 |
| 2008 | 7 | 22.2 | 11.3 | 16.8 | 5.4 |
| 2008 | 8 | 22.8 | 11.0 | 16.9 | 5.0 |
| 2008 | 9 | 23.8 | 11.4 | 17.6 | 6.2 |
| 2008 | 10 | 23.1 | 11.1 | 17.1 | 9.0 |
| 2008 | 11 | 23.5 | 11.2 | 17.4 | 5.6 |
| 2008 | 12 | 25.5 | 10.4 | 17.9 | 1.2 |
| 2009 | 1 | 25.5 | 10.8 | 18.2 | 4.4 |
| 2009 | 2 | 26.5 | 9.7 | 18.1 | 1.6 |
| 2009 | 3 | 27.6 | 11.2 | 19.4 | 2.3 |
| 2009 | 4 | 24.6 | 11.8 | 18.2 | 6.7 |
| 2009 | 5 | 23.5 | 11.7 | 17.6 | 4.1 |
| 2009 | 6 | 24.5 | 10.2 | 17.4 | 2.5 |
| 2009 | 7 | 24.0 | 10.5 | 17.3 | 2.6 |
| 2009 | 8 | 24.3 | 11.6 | 18.0 | 5.2 |
| 2009 | 9 | 24.4 | 11.7 | 18.1 | 6.4 |
| 2009 | 10 | 24.1 | 11.7 | 17.9 | 4.3 |
| 2009 | 11 | 24.6 | 11.0 | 17.8 | 3.7 |
| 2009 | 12 | 24.0 | 11.4 | 17.7 | 9.4 |
